# Experimental Investigation of Nickel-Based Co-Catalysts for Photoelectrochemical Water Splitting Using Hematite and Cupric Oxide Nanostructured Electrodes

**DOI:** 10.3390/nano15201551

**Published:** 2025-10-11

**Authors:** Maria Aurora Mancuso, Rossana Giaquinta, Carmine Arnese, Patrizia Frontera, Anastasia Macario, Angela Malara, Stefano Trocino

**Affiliations:** 1 Institute for Advanced Energy Technologies “Nicola Giordano” CNR-ITAE, Via Salita S. Lucia Sopra Contesse, 5, 98126 Messina, Italy; mariaauroramancuso@cnr.it (M.A.M.); rossanagiaquinta@cnr.it (R.G.); carmine.arnese@cnr.it (C.A.); 2 Department of Civil, Energy, Environmental and Material Engineering, Mediterranea University of Reggio Calabria, 89124 Reggio Calabria, Italy; patrizia.frontera@unirc.it (P.F.); angela.malara@unirc.it (A.M.); 3 Department of Environmental Engineering, University of Calabria, 87036 Arcavacata di Rende, Italy; anastasia.macario@unical.it

**Keywords:** co-catalysts, green hydrogen, photoelectrochemical cell, hematite, cupric oxide

## Abstract

Growing interest in sustainable hydrogen production has brought renewed attention to photoelectrochemical (PEC) water splitting as a promising route for direct solar-to-chemical energy conversion. This study explores how integrating hematite (α-Fe_2_O_3_) and cupric oxide (CuO) photoelectrodes with a series of nickel-based co-catalysts can improve photoelectrochemical activity. Photoanodic (NiO_x_, NiFeO_x_, NiWO_4_) and photocathodic (Ni, NiCu, NiMo) co-catalysts were synthesized via co-precipitation and mechanochemical methods and characterized through X-ray Diffraction (XRD), X-ray Fluorescence (XRF), Transmission Electron Microscopy–Energy Dispersive X-ray Spectroscopy (TEM-EDX), Scanning Electron Microscopy–Energy Dispersive X-ray Spectroscopy (SEM-EDX), X-ray photoelectron spectroscopy (XPS) and Brunauer–Emmett–Teller (BET) gas-adsorption analyses to clarify their crystallographic, morphological, and compositional properties, as well as their surface chemistry and textural properties (surface area and porosity). Electrochemical tests under 1 SUN illumination showed that NiO_x_ significantly improves the photocurrent of hematite photoanodes. Among the cathodic co-catalysts, NiMo demonstrated the best performance when combined with CuO photocathodes. For both photoelectrodes, an optimal co-catalyst loading was identified, beyond which performance declined due to potential charge transfer limitations and light attenuation. These findings highlight the critical role of co-catalyst composition and loading in optimizing the efficiency of PEC systems based on earth-abundant materials, offering a pathway toward scalable and cost-effective hydrogen production.

## 1. Introduction

The increasing demand for sustainable and clean energy sources has driven significant interest in green hydrogen production technologies as a cornerstone for deep decarbonization across industry, transport, and the power sector [1,2]. Among the available routes, photoelectrochemical (PEC) water splitting has emerged as a particularly promising approach because it directly converts solar energy into chemical energy stored in hydrogen and oxygen through the splitting of water [3,4,5,6,7,8]. In a PEC cell, a semiconductor photoelectrode absorbs sunlight and generates electron–hole pairs that must be separated, transported, and transferred to the electrolyte to drive the half-reactions of water oxidation (OER) and proton reduction (HER). To accomplish this efficiently, the photoelectrode must unite intrinsic optical absorption in the visible range, sufficiently long carrier lifetimes and diffusion lengths, and favorable interfacial charge-transfer kinetics at the solid–liquid boundary [9,10,11,12]. In addition, the band-edge positions must straddle the water redox potentials to enable thermodynamically feasible reactions under illumination, while chemical stability in aqueous environments, abundance, and low cost remain essential for scalability [9,10,11,12]. These multiple, often competing, requirements motivate materials design strategies that combine appropriate semiconductor absorbers with surface co-catalysts capable of mitigating kinetic bottlenecks and suppressing surface recombination.

Within this framework, hematite (α-Fe_2_O_3_) has been widely investigated as an n-type photoanode thanks to its abundance, environmental compatibility, and stability in alkaline electrolytes. Its bandgap of 2.1–2.2 eV enables absorption of a substantial portion of the solar spectrum up to ~590–620 nm, and the valence band is suitably positioned for OER, which makes hematite particularly appealing for the oxidative half-reaction [12,13,14]. Nonetheless, its conduction band is not ideally placed for proton reduction, and the material suffers from short carrier diffusion lengths and slow surface kinetics, frequently requiring an external bias or complementary device concepts to reach practical efficiencies [15,16,17,18]. On the photocathode side, cupric oxide (CuO) is a p-type semiconductor with a narrow bandgap (~1.2–1.7 eV) that ensures strong visible-light absorption and band-edge positions compatible with HER, while also offering advantages of earth abundance and low cost [19,20].

**Figure 1:**
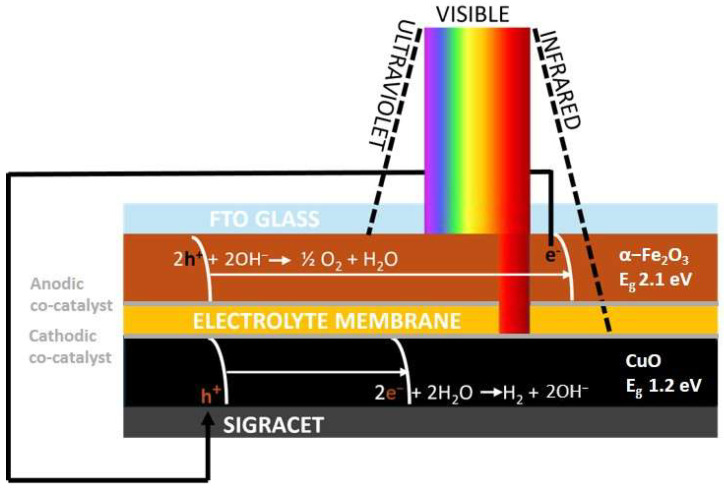
Schematic representation of photoelectrochemical water splitting process.

A well-established strategy to circumvent the inherent limitations of single absorbers is the tandem PEC configuration, where two semiconductors operate synergistically as photoanode and photocathode. In this architecture, both electrodes absorb solar light and generate charges: holes at the photoanode drive OER, whereas electrons at the photocathode sustain HER. In the photoanode, band-edge bending favors hole transport toward the electrolyte and requires a valence band sufficiently positive to drive oxygen evolution; in the photocathode, band-edge bending promotes electron transfer to the electrolyte, requiring a conduction band sufficiently negative for hydrogen evolution. In our layout, hematite, directly exposed to solar irradiation, absorbs higher-energy photons in line with its bandgap (~2.1 eV), while the underlying CuO, illuminated by transmitted or diffusely scattered light through the electrolyte, harvests lower-energy photons consistent with its narrower gap (~1.2–1.7 eV) (Figure 1).

This spectral complementarity broadens solar utilization and can reduce or even eliminate the need for external bias. Nevertheless, photostability, bulk/surface recombination, and sluggish interfacial kinetics still limit attainable efficiencies and motivate surface engineering via co-catalyst integration [17,18,19,20,21].

To address kinetic limitations at the semiconductor/electrolyte interface, numerous studies have explored surface co-catalysts capable of accelerating charge transfer and stabilizing reaction intermediates. In this context, nickel-based materials have proven particularly attractive for both OER at the photoanode and HER at the photocathode because they are earth-abundant, cost-effective, chemically robust in alkaline media, and electrocatalytically active [19,20,22,23]. Nickel oxides/hydroxides and their mixed phases are known to form Ni(OH)_2_/NiOOH-like redox couples under anodic polarization that facilitate hole transfer during OER, while metallic/alloyed Ni systems (e.g., with Cu or Mo) can optimize hydrogen adsorption energetics and enhance electronic conductivity for HER. Despite this broad literature, two important gaps remain: (i) there is a lack of systematic, side-by-side comparisons of multiple Ni-based co-catalysts applied to both n-type and p-type semiconductor electrodes under identical synthesis, loading, and testing protocols; and (ii) the influence of co-catalyst loading—frequently showing volcano-type trends due to the interplay between active-site density, light shading, and transport effects—has not been consistently quantified in PEC configurations. Addressing these gaps is essential to disentangle geometric from electronic factors and to identify general descriptors linking surface composition/structure to interfacial charge-transfer kinetics and photoresponse.

Building on these premises, this work focuses on α-Fe_2_O_3_ photoanodes and CuO photocathodes modified with Ni-based co-catalysts, with the dual goal of (i) establishing comparative activity trends across oxide-type and metallic/alloy systems and (ii) quantifying the impact of co-catalyst loading under consistent conditions.

Specifically, we address:

- Q1: How do crystallographic, morphological, and surface features of selected Ni-based co-catalysts correlate with OER on Fe_2_O_3_ and HER on CuO under identical preparation and testing protocols?
- Q2: What relative activity trends emerge when comparing oxide-type (e.g., NiO_x_, NiFeO_x_) versus metallic/alloy co-catalysts (e.g., Ni, NiCu, NiMo) on n-type and p-type photoelectrodes?
- Q3: How does co-catalyst loading modulate performance and does it manifest a volcano-type dependence, revealing optimal ranges tied to active-site availability and transport/optical constraints?

To answer these questions, we present—for the first time in a controlled half-cell comparative framework—a side-by-side evaluation of multiple Ni-based co-catalyst compositions and loadings on both photoanodes (Fe_2_O_3_) and photocathodes (CuO). The study integrates structural (XRD), morphological/compositional (SEM/TEM-EDX, XRF), and surface-chemistry/textural analyses (XPS, BET) with photoelectrochemical measurements under 1 SUN, enabling a direct correlation between surface and textural properties, interfacial charge-transfer characteristics, and the PEC response. This systematic approach provides a quantitative baseline to rationalize the role of Ni-based co-catalysts in Fe_2_O_3_/CuO tandem-oriented architectures and to guide materials selection and loading optimization for green hydrogen production via photoelectrolysis.

Within this screening scope, long-term durability is not addressed and will be assessed in future work using full Fe_2_O/CuO tandem cells under device-relevant operating protocols.

## 2. Materials and Methods

### 2.1. Ni-Based Co-Catalysts Development

Materials such as NiO_x_, NiFeO_x_, and NiWO_4_ have been proposed as anodic co-catalysts for the oxygen evolution reaction (OER), while Ni, NiCu, and NiMo have demonstrated effectiveness as cathodic co-catalysts for the hydrogen evolution reaction (HER). These co-catalysts have been synthesized using co-precipitation and solid-state methods.

#### 2.1.1. Synthesis of NiO_x_ and Ni

For the synthesis of NiO_x_ and metallic Ni co-catalysts, Ni(NO_3_)_2_·6H_2_O (Sigma-Aldrich, St. Louis, MO, USA) was solubilized in 100 mL of pure water. The solution was placed in a hot water bath maintained at 60 °C, and the pH was gradually adjusted to 9 by adding 2 M NaOH dropwise. After having reached the desired pH, the solution was kept under magnetic stirring for 3 h. The precipitate obtained was filtered and then dried overnight at 80 °C. Part of the dried powder was calcined at 350 °C for 2 h to promote the formation of NiO_x_ and was ground using a pestle and mortar, while the remaining part was reduced at 330 °C for 30 min to obtain metallic nickel. A schematic representation of the synthesis process is shown in Figure S1.

#### 2.1.2. Synthesis of NiFeO_x_

NiFeO_x_ was obtained by co-precipitation method [21,23]. Ni(NO_3_)_2_·6H_2_O and Fe(NO_3_)_2_·9H_2_O (Sigma-Aldrich, St. Louis, MO, USA) were used as starting materials. Both precursors were dissolved in 50 mL of distilled water and placed in a hot water bath at 60 °C. The process scheme is shown in Figure S2. After measuring the initial pH, a 2 M NaOH solution was slowly added until the pH reached 10, while stirring continuously for 4 h. The resulting precipitate was then filtered, washed with 250 mL of pure water, and dried at 80 °C for 12 h.

#### 2.1.3. Synthesis of NiWO_4_

To directly synthesize NiWO_4_, a mechanochemical activation followed by calcination has been used [24]. A stoichiometric mixture of NiO and WO_3_ in a 1:1 molar ratio was subjected to mechanical treatment in air using ball milling for 10 h at 450 rpm at room temperature. Afterwards, the powder was calcined at 850 °C for 7.5 h producing a yellow powder. This approach avoids both complex procedures and unwanted sintering, which would otherwise lead to particle agglomeration and a dense, compact structure. Avoiding sintering is crucial to preserving the powdery morphology and maintaining a high surface area for catalytic applications. The synthesis process is schematically depicted in Figure S3.

#### 2.1.4. Synthesis of NiCu

The NiCu co-catalyst was obtained by chemical co-precipitation from nickel and copper nitrate salts. Nickel nitrate hexahydrate (Ni(NO_3_)_2_·6H_2_O) (Sigma-Aldrich, St. Louis, MO, USA) and copper nitrate hemihydrate (Cu(NO_3_)_2_)·1/2H_2_O (Sigma-Aldrich, St. Louis, MO, USA) were used as precursors in a 1:1 molar ratio and dissolved in 100 mL of pure water. A water bath was heated to 60 °C to maintain a constant temperature during the reaction. The initial pH was adjusted to 9 by adding a 2 M NaOH solution. Once the desired pH was reached, the solution was stirred magnetically for 4 h. The resulting precipitate was filtered, washed with distilled water and dried overnight at 80 °C. Finally, reduction was carried out at 350 °C for 30 min to promote the formation of NiCu. A schematic overview of the synthesis is provided in Figure S4 [20].

#### 2.1.5. Synthesis of NiMo

NiMo alloy was synthesized by co-precipitation method [25]. Nickel Nitrate Hexahydrate (Ni(NO_3_)_2_∙6H_2_O) (Sigma-Aldrich, St. Louis, MO, USA) and ammonium molybdate tetrahydrate ((NH_4_)_6_Mo_7_O_24_∙4H_2_O) (Sigma-Aldrich, St. Louis, MO, USA) were dissolved in ultrapure distilled water. The solution was placed in a hot water bath maintained at 60 °C and stirred. When the temperature was reached, the pH was corrected with a solution of 2 M NaOH until pH = 11. The solution was maintained at 60 °C for 6 h. Afterwards, the precipitate was filtered and washed with distilled water and dried at 80 °C overnight.

The reduction occurred at 550 °C for 6 h to allow the formation of NiMo alloy. The synthesis process is schematically reported in Figure S5.

### 2.2. Physico-Chemical Characterization

The physico-chemical and morphological characteristics of the co-catalysts were investigated using X-ray diffraction (XRD), X-ray fluorescence (XRF), scanning electron microscopy with energy-dispersive X-ray spectroscopy (SEM-EDX), transmission electron microscopy with EDX (TEM-EDX), X-ray photoelectron spectroscopy (XPS), and Brunauer–Emmett–Teller (BET) gas-adsorption analysis.

A Bruker (Bruker AXS Inc., Madison, WI, USA) D2 PHASER desktop diffractometer was used for investigating the crystallographic phases and the crystallite size of the synthesized co-catalysts. The measurements were carried out in Bragg-Brentano geometry, operating with a Ni b-filtered Cu-Kα radiation (λ = 1.5406 Å) in the 2θ range 5–100° at 40 kV and 40 mA and a scan step of 0.03° s^−1^. The diffraction patterns were analyzed through the Joint Committee on Powder Diffraction Standards (JCPDS).

The co-catalyst elemental composition was determined by XRF investigations using a S8 TIGER spectrometer (Bruker AXS Inc., Madison, WI, USA), equipped with a rhodium anode tube (power 4 kW and 75 µm Be window and LiF 220 crystal analyze).

TEM analysis was performed using a JEOL JEM-F200 (Jeol Italia, Milan, Italy) microscope equipped with an EDX detector to examine the morphology and elemental distribution of the electrocatalysts. The samples were prepared by ultrasonic dispersion of the catalyst in isopropyl alcohol and depositing then a drop of the suspension on a holey carbon–coated Cu grid. SEM analyses were performed using a FEI S-FEG XL30 microscope (FEI Company, Hillsboro, OR, USA) equipped with an EDX spectrometer (FEI Company, Hillsboro, OR, USA), operating at 25 kV.

The surface elemental composition of catalysts were studied by X-ray photoelectron spectroscopy (XPS). This was carried out using a Physical Electronics (PHI) 5800–01 (Physical Electronics, MN, USA) spectrometer equipped with a monochromatic Al Kα X-ray source.

To complete the physico-chemical characterization, specific surface area and porosity were measured by gas adsorption using a Gemini surface area analyzer (Micromeritics Instrument Corporation, Norcross, GA, USA). Before measurements, powders were degassed under vacuum at 150 °C for 4 h to remove physisorbed species. Nitrogen adsorption–desorption at 77 K (−196 °C) was employed for the oxide samples (NiO_x_, NiFeO_x_, NiWO_4_), whereas, due to their very low surface area, the metallic samples (Ni, NiCu, NiMo) were analyzed in krypton adsorption mode at 77 K. BET specific surface area was calculated in the relative pressure range 0.05–0.30 (N_2_) and 0.05–0.25 (Kr), while the pore-size distribution of the oxides was obtained by the Barrett–Joyner–Halenda method (BJH desorption branch).

### 2.3. Preparation of Semiconductors and Co-Catalysts Deposition

Hematite (α-Fe_2_O_3_) photoanodes were prepared by the following synthetic procedure [18]. Vertically aligned FTO substrates—partially covered with adhesive tape to allow electrical contact—were placed in a sealed glass reactor containing an aqueous solution of 0.15 M FeCl_3_ and 1 M NaNO_3_. The chemical bath deposition was carried out by heating the reactor at 100 °C for 6 h. After the reaction, the electrodes were rinsed with deionized water and dried at room temperature, resulting in the formation of FeOOH on the FTO surface. Finally, the electrodes were thermally annealed in air at 650 °C for 1 h to convert FeOOH into α-Fe_2_O_3_.

The CuO precursor powder was synthesized using the oxalate-based route. The powder was subjected to a thermal treatment at 350 °C for 2 h to obtain CuO nanoparticles. In order to enhance film uniformity and adhesion, a subsequent reduction step was carried out: the powder was exposed to a 10% H_2_–90% N_2_ gas flow at 200 °C, resulting in the formation of finely dispersed metallic copper. The sample was then left to cool overnight under an inert atmosphere. The resulting copper particles were ultrasonically dispersed in isopropyl alcohol and spray-deposited onto substrates with a defined active area of 1 cm^2^ (geometric area). The cathodic substrates employed was Sigracet 35BC^®^. A metal loading of 2.0 mg cm^−2^ was achieved. To regenerate the CuO phase, the electrodes were finally annealed in air at 350 °C. The intermediate reduction to metallic Cu was a crucial step to promote uniform coverage and enhance the adhesion of the CuO layer to the substrate surface [18].

Co-catalysts were deposited onto the photoanode by drop-casting a solution of the specific co-catalyst in isopropyl alcohol. Following the deposition, the electrodes underwent a thermal treatment at 450 °C for 1 h to ensure proper adhesion and activation of the co-catalyst layer.

Similarly, co-catalysts for the photocathode were applied via drop-casting of their solution in isopropyl alcohol, with a subsequent thermal annealing at 300 °C for 1 h.

### 2.4. Half-Cell Characterization

The photoactivity of the electrodes was investigated in a half-cell configuration, using a sealed plastic reactor under N_2_-saturated conditions. The conventional three-electrode setup consisted of a saturated Ag/AgCl reference electrode (3 M KCl), a platinum wire counter electrode, and the photoelectrode under study as the working electrode. Electrochemical tests were carried out by measuring the current at varying potential with respect to the Ag/AgCl reference electrode. The experimental setup shown in Figure S6 was used for electrochemical characterization.

The polarization curves were measured in the dark and under illumination with a scan rate of 20 mV/s. These measurements were conducted using a potentiostat/galvanostat equipped with a frequency response analyzer from Metrohm (Autolab PGSTAT 302N, Metrohm Autolab B.V., Utrecht, The Netherlands).

Photoanode characterization was performed in 1 M KOH under nitrogen flow using a solar simulator (Oriel Sol3A Class AAA, Newport Corporation, Irvine, CA, USA) calibrated to 1 SUN AM 1.5 (100 mW cm^−2^), by scanning the potential from 0 to 0.4 V vs. Ag/AgCl.

Photocathodes were characterized under the same conditions, by scanning the potential from −0.4 V to 0 V vs. Ag/AgCl.

Photocurrent density was calculated as the difference between the light and dark current densities.

### 2.5. Electrochemical Surface Area (ECSA) Determination

ECSA was estimated from the double-layer capacitance (*C_dl_*) obtained by cyclic voltammetry (CV) in the non-faradaic region, using the same three-electrode half-cell and potentiostat adopted for polarization curves (Metrohm Autolab PGSTAT302N, Metrohm Autolab B.V., Utrecht, The Netherlands). Measurements were carried out in 1 M KOH, N_2_-saturated, in the dark to exclude photogenerated contributions. The potential windows were selected to avoid redox features of the substrates/co-catalysts and gas-evolution onsets:

- Photoanodes: from 0.05 to 0.15 V vs. Ag/AgCl, scan rates 10–100 mV s^−1^.
- Photocathodes: from −0.15 to −0.05 V vs. Ag/AgCl, scan rates 10–100 mV s^−1^.

For each scan rate, the capacitive current density (j_cap_) was taken at the window midpoint and the slope of j_cap_ vs. scan rate yielded *C_dl_*. *ECSA* was then computed as

$$ECSA=\frac{C_{dl}}{C_{s}}\times A_{geo}$$

assuming *A_geo_* = 1 cm^2^ and a specific capacitance *C_s_* = 40 µF cm^−2^ in alkaline media for oxide/metallic surfaces; this value is widely adopted for comparing nanostructured electrodes.

## 3. Results

### 3.1. Physico-Chemical Characterization of Co-Catalysts by XRD and XRF Analysis

#### 3.1.1. Physico-Chemical Properties of NiO_x_

**Figure 2:**
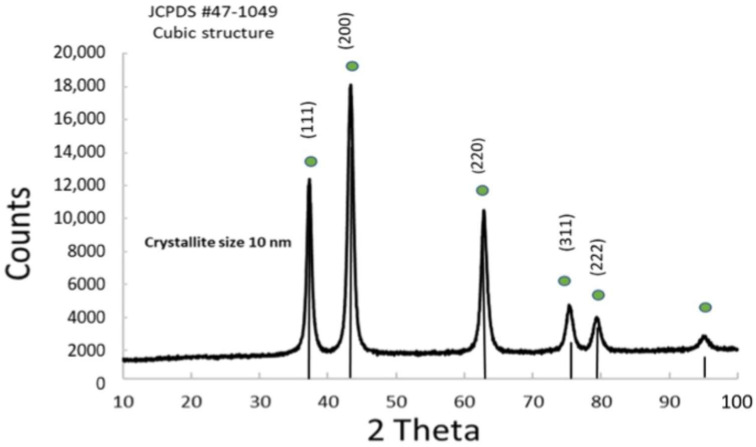
XRD pattern of the anodic NiO_x_ co-catalyst.

The obtained powders were analyzed through XRD. The XRD spectrum of NiO_x_ (Figure 2) revealed well-defined peaks, indicative of a crystalline morphology, with an average crystallite size of 10 nm, calculated using the Scherrer formula. The precursor Ni(OH)_2_ was subjected to calcination, leading to the formation of peaks corresponding to NiO_x_ (Figure S1). The diffraction pattern confirms that NiO_x_ crystallizes in a cubic structure, consistent with (JCPDS #47-1049) [26]. Elemental composition was assessed through XRF analysis to provide semi-quantitative data, revealing a NiO_x_ without impurities from the synthesis. Figure S7 reports the characteristic x-ray emissions from the K-alpha and K-beta lines of Ni.

#### 3.1.2. Physico-Chemical Properties of NiFeO_x_

**Figure 3:**
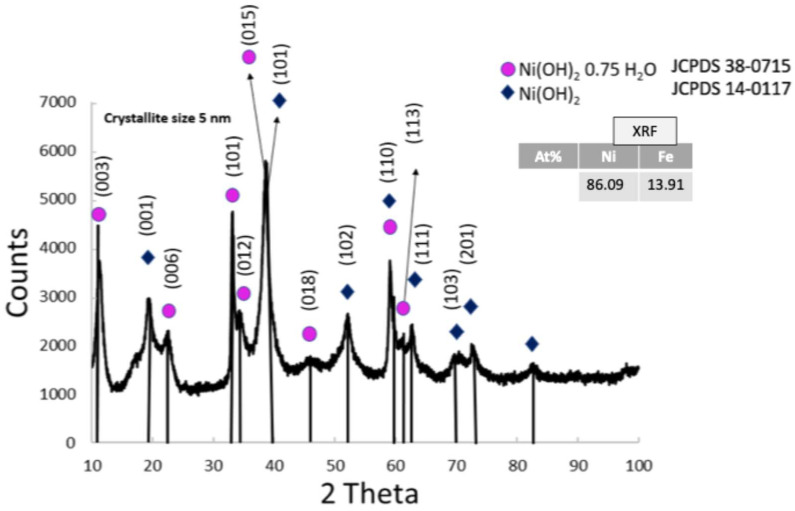
XRD pattern of the anodic NiFeO_x_ co-catalyst and the atomic ratio from XRF analysis in the inset.

Figure 3 presents the XRD pattern of the synthesized anode co-catalyst, which indicates the presence of two phases, α-Ni(OH)_2_·0.75H_2_O (JCPDS 38-0715) with a rhombohedral crystal structure, and Ni(OH)_2_ (JCPDS 14-0117), consistently with previous papers [21,23,27,28]. Although no XRD peaks related to iron (Fe) are observed, this is due to the fact that Fe was alloyed with Ni, resulting in a more stable and homogeneous structure where Fe was atomically dispersed and thus undetectable by XRD. The average crystallite size was determined to be around 5 nm using the Scherrer equation. As shown in the inset of Figure 3, XRF analysis revealed an atomic ratio of 86.09% nickel and 13.91%. Figure S8 shows the characteristic x-ray emission lines Kα and Kβ for the elements Ni and Fe.

#### 3.1.3. Physico-Chemical Properties of NiWO_4_

**Figure 4:**
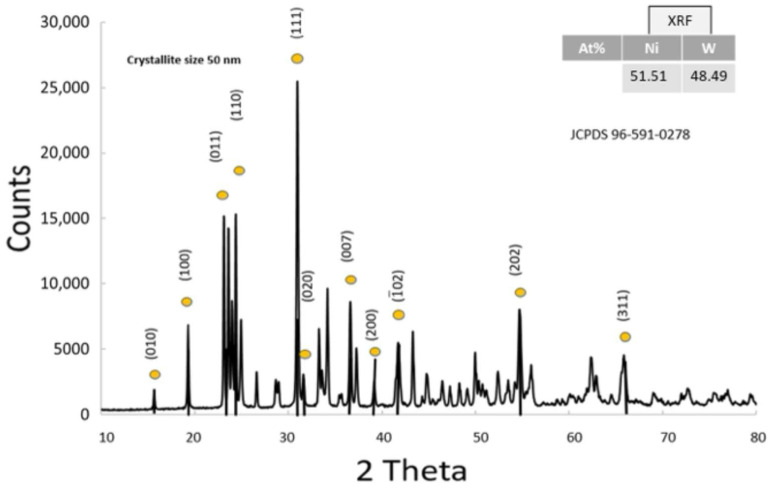
XRD pattern of the anodic NiWO_4_ co-catalyst and the atomic ratio from XRF analysis in the inset.

The XRD pattern of NiWO_4_ exhibits characteristic peaks corresponding to its monoclinic wolframite-type structure [29] (JCPDS 96-591-0278). The pattern shows well-defined, sharp peaks, indicating a highly crystalline nature (Figure 4). Using the Scherrer equation, the average crystallite size was calculated to be 50 nm. X-ray fluorescence analysis exhibits characteristic K-alpha and K-beta lines of nickel and K-alpha, K-bea, L-alpha and L-beta lines of tungsten as shown in Figure S9. NiWO_4_ atomic percentages were calculated as Ni = 51.51% and W = 48.49%.

#### 3.1.4. Physico-Chemical Properties of Ni

**Figure 5:**
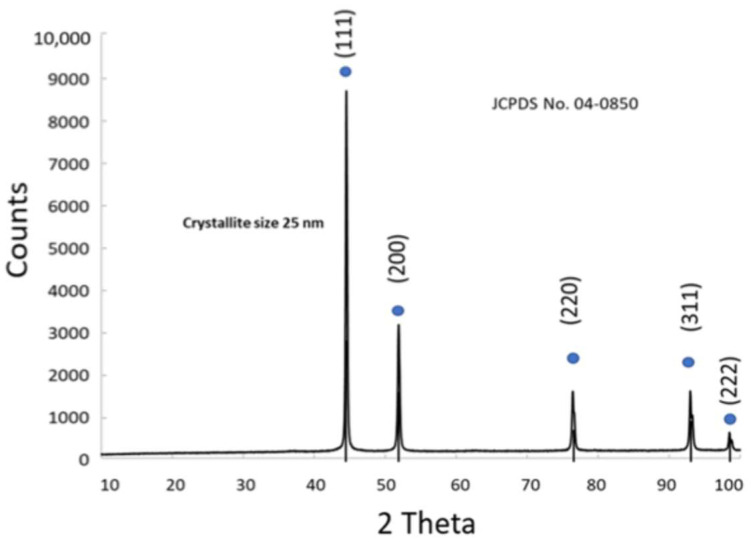
XRD pattern of the cathodic Ni co-catalyst and the XRF in the inset.

The diffractogram of resulting Ni powder shows narrow peaks at 2ϴ = 44, 52, 76, 93, 99° (JCPDS 04-0850) [30] (Figure 5). The average crystallite size was 25 nm. The atomic composition of the cathodic Ni co-catalyst was determined by XRF analysis and the typical K-alpha and K-beta lines of Ni are reported in Figure S10.

#### 3.1.5. Physico-Chemical Properties of NiCu

**Figure 6:**
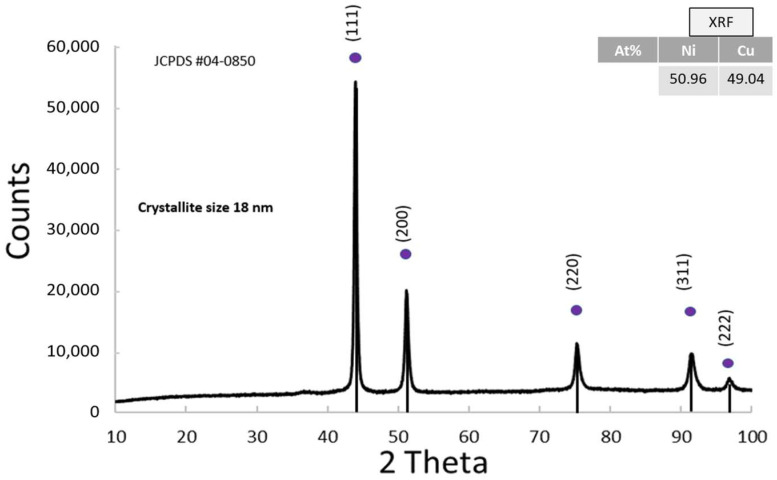
XRD pattern of the cathodic co-catalyst NiCu and the atomic ratio from XRF analysis in the inset.

The XRD pattern of the NiCu alloy confirms that the material has a face-centered cubic (FCC) structure (JCPDS 04-0850). The presence of very small crystallites results in broadened diffraction peaks (Figure 6). The absence of secondary phases in the XRD pattern suggests that the alloy is a single-phase system, meaning that Ni and Cu atoms are uniformly distributed within the lattice without phase segregation. Although both nickel and copper are present in nearly equiatomic proportions, no distinct peaks related to pure Cu are observed. This is expected, as Ni and Cu are completely miscible in the solid state and share the same FCC structure with very similar lattice parameters. As a result, the diffraction peaks correspond to a solid alloy where the Cu atoms substitute Ni atoms in the lattice sites, causing only slight shifts in peak positions.

X-ray fluorescence analysis revealed the presence of both K-alpha and K-beta emission lines characteristic of nickel and copper, as illustrated in Figure S11. The NiCu alloy had a 1:1 molar ratio, which corresponds to an atomic composition of Ni = 50.96% and Cu = 49.04%.

#### 3.1.6. Physico-Chemical Properties of NiMo

**Figure 7:**
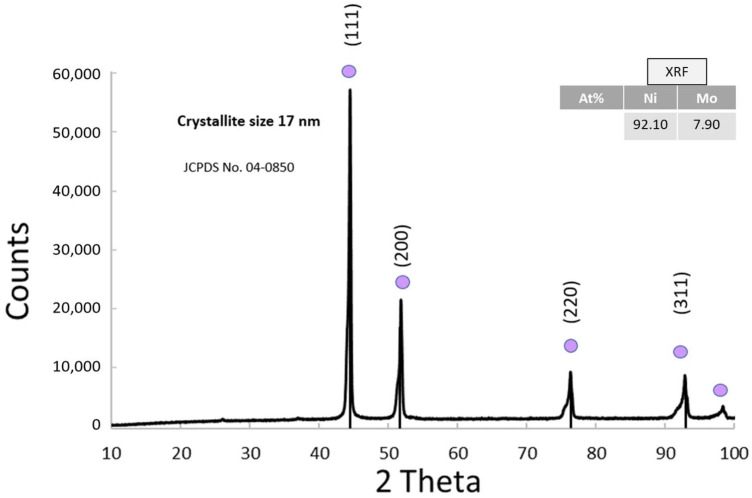
XRD pattern of the cathodic co-catalyst NiMo and the atomic ratio from XRF analysis in the inset.

After reducing the NiMo co-catalyst, XRD analysis revealed a typical nickel’s pattern with narrow peaks (JCPDS 04-0850) (Figure 7); through the Scherrer formula it was possible to calculate an average crystallite size of 17 nm. Reduction of NiMo oxide significantly altered the crystal structure. For example, XRD analysis does not show visible peaks for Mo, suggesting that molybdenum was incorporated at the atomic level. This is because Mo alloyed with Ni, forming a structure where it was distributed at the atomic level and therefore not detectable by XRD. The atomic percentage obtained by XRF analysis revealed the more abundance of Ni (92.10%) compared to Mo (7.90%). Indeed, in Figure S12, the characteristic lines K-alpha and K-beta of Ni as well as those of Mo are shown.

### 3.2. Physico-Chemical Characterization of Co-Catalysts by TEM-EDX and SEM-EDX Analysis

**Figure 8:**
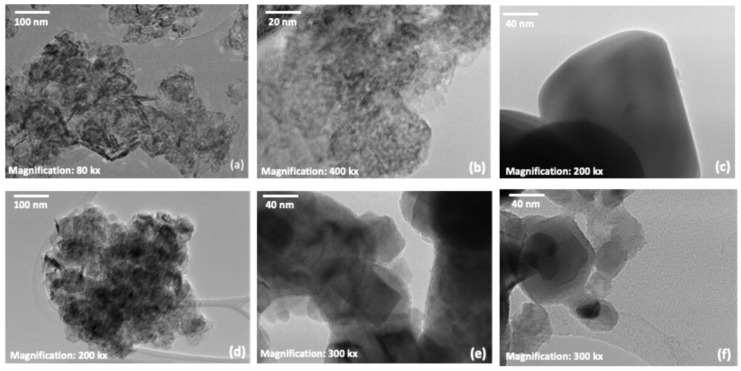
TEM image of co-catalysts: NiO_x_ (**a**), NiFeO_x_ (**b**), NiWO_4_ (**c**), Ni (**d**), NiCu (**e**) NiMo (**f**).

For the NiO_x_ sample (Figure 8a), TEM images at 80 kx magnification reveal a morphology composed of elongated-shaped clusters and smaller particles, with sizes ranging from 10 to 14 nm. These values are consistent with the crystallite size estimated from XRD using the Scherrer formula (Figure 2).

The NiFeO_x_ sample (Figure 8b) displayed a morphology consisting of a mixture of circular nanoparticles with diameters smaller than 3 nm, as observed at 400 kx of magnification. EDX analysis in Figure S13a confirms the presence of nickel, iron, and oxygen, with atomic percentages of 30.99%, 5.18%, and 63.83%, respectively.

In Figure 8c is reported TEM images taken at 200 kx magnification for NiWO_4_, showing cubical-shaped particles. Elemental mapping by EDX (Figure S13b) confirmed the expected composition, detecting 15.04% Ni, 14.03% W, and 70.93% O, consistent with NiWO_4_ stoichiometry.

The Ni sample in Figure 8d, imaged at 80 kx magnification, exhibited a morphology composed of regular polygonal clusters. EDX analysis confirmed the exclusive presence of Ni, as also shown in the table in Figure S13c.

In the case of the NiCu sample (Figure 8e), observed at 300 kx magnification, the particles exhibited a regular, squared shape. Most of them were under 4 nm in size. The corresponding EDX analysis (Figure S13d) confirmed the presence of both Ni and Cu.

Lastly, TEM images of NiMo (Figure 8f), also acquired at 300 kx magnification, show a mixed morphology composed of small circular and square-like nanoparticles, with average sizes around 20 nm. According to EDX analysis (Figure S13e), the atomic composition was 93.84% Ni and 6.16% Mo, in good agreement with XRF measurements.

**Figure 9:**
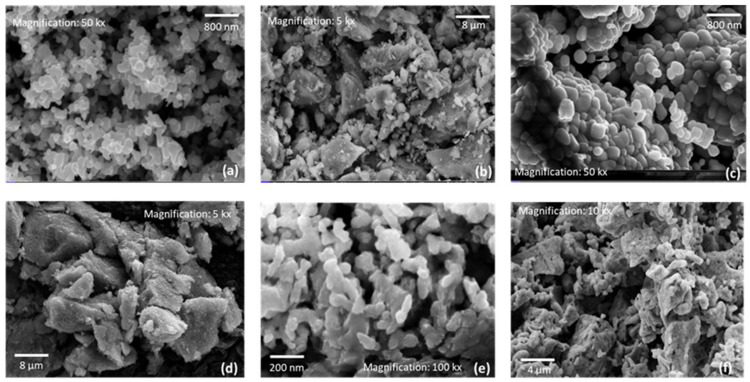
SEM image of co-catalysts: NiO (**a**), NiFeO_x_ (**b**), NiWO_4_ (**c**), Ni (**d**), NiCu (**e**) NiMo (**f**).

The SEM image of the NiO_x_ sample (Figure 9a) reveals a mixture of irregularly shaped particles. As shown in the corresponding TEM image (Figure 8a), the NiO_x_ nanocrystals were organized into rectangular structures. EDX analysis (Figure S14a) confirmed the elemental composition, in agreement with XRF and XRD results.

In the case of the NiFeO_x_ sample (Figure 9b), the SEM image displays a combination of circular and irregularly shaped particles. EDX analysis (Figure S14b) provided atomic percentages of Ni (42.6%), Fe (4.8%), and O (52.6%), closely matching the values obtained from TEM-based measurements.

The NiWO_4_ sample (Figure 9c) exhibited mostly cubic and well-defined particles in the SEM image, consistent with the morphology observed in the TEM image (Figure 8c). Elemental composition from EDX is reported in Figure S14c.

For the Ni sample (Figure 9d), SEM analysis showed regular polygonal particles, which aligned well with the morphology seen in TEM images. EDX results are presented in Figure S14d.

The SEM image of NiCu (Figure 9e) shows a uniform particle distribution. EDX analysis (Figure S14e) revealed a near-equimolar composition of Ni (49.1%) and Cu (50.9%), consistent with XRF data and confirming the intended stoichiometric ratio.

Lastly, the NiMo sample (Figure 9f) displayed irregular surfaces, and particles with rounded edges were observed, a typical feature of co-precipitation followed by thermal treatment. No well-defined crystalline structures are observed, suggesting a predominantly amorphous or nanocrystalline nature. EDX analysis indicated atomic percentages of Ni (99.2%) and Mo (0.8%) (Figure S14f), which were in good agreement with both TEM-EDX and XRF measurements, within the acceptable margin of error.

### 3.3. Physico-Chemical Characterization of Co-Catalysts by Brunauer–Emmett–Teller (BET) Analysis

Gas-adsorption measurements highlight a clear textural contrast between oxide and metallic/alloy co-catalysts. The oxide powders (NiO_x_, NiFeO_x_, NiWO_4_) display type-IV isotherms with H3/H4 hysteresis, consistent with mesoporosity. Their BJH pore-size distributions (desorption branch) show modal diameters in the 6–12 nm range, in agreement with the aggregated nanocrystallites observed by TEM/SEM. In contrast, the metallic/alloy samples (Ni, NiCu, NiMo), analyzed in Kr @77 K mode due to their very low surface area, exhibit near type-II behavior with negligible pore volume, indicative of essentially non-porous surfaces. Among the oxides, NiO_x_ shows the largest specific surface area and mesopore volume, followed by NiFeO_x_ and NiWO_4_. All metallic/alloy powders present specific surface area <1 m^2^ g^−1^ with vanishing porosity. Overall, these results indicate that, while oxide texturing can increase the number of accessible interfacial sites, geometric surface area alone does not govern PEC performance—as discussed later for NiMo on CuO, where superior photocurrents are obtained despite very low specific surface area, pointing to a dominant role of electronic/interfacial kinetics. The principal descriptors are summarized in Table 1 and Table 2.

### 3.4. Physico-Chemical Characterization of Co-Catalysts by XPS Survey Analysis

The XPS survey spectra (Figure S15) reveal the presence of the expected elements on the surface of each sample: Ni and O in NiO_x_; Ni, Fe, and O in NiFeO_x_; Ni, W, and O in NiWO_4_; metallic Ni with only minor surface oxidation; Ni and Cu in the NiCu alloy; and Ni, Mo, and O in the NiMo-based catalyst. No extraneous signals were detected above the background, confirming the chemical purity of the materials. These results demonstrate a close consistency among the different techniques, with surface-sensitive XPS complementing bulk-sensitive XRD and EDX/XRF analyses.

### 3.5. Electrochemical Characterization of Electrodes

An electrochemical comparison, in half-cell configuration, was made among the different Ni-based co-catalysts deposited on the hematite (Fe_2_O_3_) and cupric oxide (CuO).

**Figure 10:**
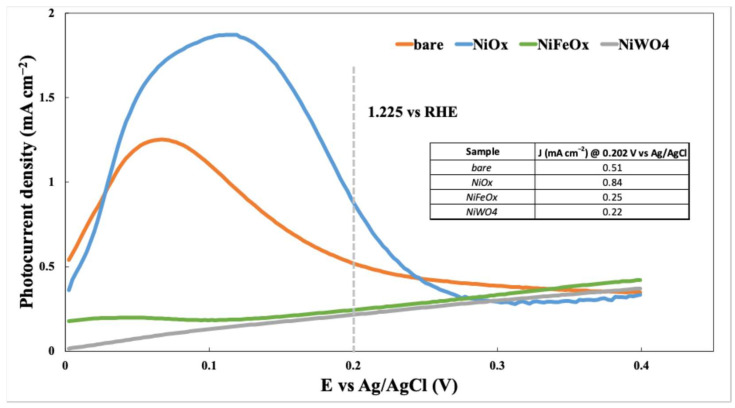
Photocurrent density vs. applied potential (vs. Ag/AgCl) for Fe_2_O_3_ photoanodes modified with Ni-based anodic co-catalysts (NiO_x_, NiFeO_x_, NiWO_4_) and the unmodified photoanode. All measurements were performed in 1 M KOH under 1 SUN AM 1.5 illumination with 16 µg cm^−2^ catalyst loading.

For the half-cell electrochemical characterization of the photoanode, by scanning the potential from 0 V to 0.4 V vs. Ag/AgCl, the following results were obtained (Figure 10), reported as the difference between the current density measured in the light and the current density measured in the dark.

Figure 10 presents the photocurrent density as a function of applied potential (vs. Ag/AgCl) for various photoanodes modified with different nickel-based co-catalysts: bare (without co-catalyst), NiO_x_, NiFeO_x_, and NiWO_4_ (all with a catalyst loading of 16 µg cm^−2^).

The figure demonstrates that NiO_x_-modified photoanode exhibits enhanced photocurrent density compared to the bare photoanode. Specifically:

- NiO_x_: This shows the most significant improvement in photocurrent density, reaching values exceeding 0.8 mA/cm^2^ at approximately 0.2 V vs. Ag/AgCl. This suggests that NiO_x_ is a highly effective co-catalyst for boosting the photoanode’s activity.
- NiFeO_x_ and NiWO_4_: Over the potential range, both deliver photocurrents that are well below those of NiO_x_ and generally below those of the bare photoanode. At 0.20 V vs. Ag/AgCl, they are lower than the bare photoanode, indicating that they do not provide an improvement under our conditions. This likely reflects less favorable surface states and interfacial charge-transfer kinetics compared to NiO_x_.

The table embedded in the graph quantifies these observations by listing the photocurrent density values at 0.202 V vs. Ag/AgCl for each sample. The trend of the curves suggests that although NiO_x_ shows the best performance, the photocurrent density reaches a plateau above ~0.3 V vs. Ag/AgCl for all co-catalysts. This could indicate a limitation in charge carrier transport within the semiconductor itself, rather than solely the co-catalyst’s catalytic activity. In conclusion, Figure 10 effectively illustrates the efficacy of NiO_x_ co-catalyst in improving the photoactivity of a Fe_2_O_3_ photoanode in a half-cell configuration.

**Figure 11:**
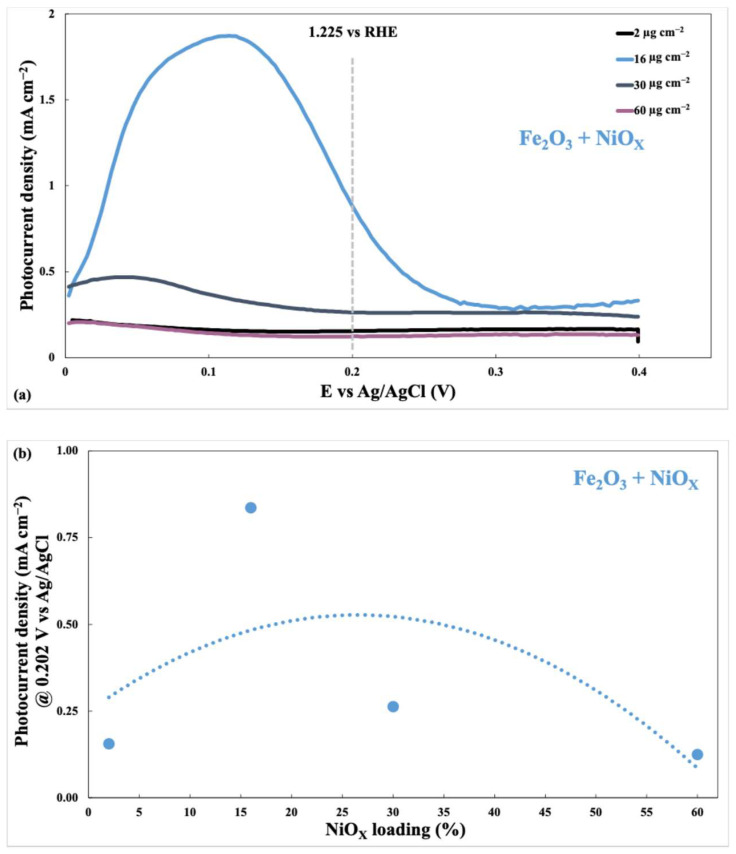
(**a**) Polarization curves of Fe_2_O_3_ photoanodes with different NiO_x_ loadings (2–60 µg cm^−2^); (**b**) Volcano trend in photocurrent density vs. NiO_x_ loading at 0.202 V vs. Ag/AgCl in 1 M KOH electrolyte under 1 SUN illumination.

Figure 11a shows the photocurrent density as a function of applied potential (vs. Ag/AgCl) for a Fe_2_O_3_ photoanode modified with varying loadings of NiO_x_ co-catalyst (2 µg cm^−2^, 16 µg cm^−2^, 30 µg cm^−2^, and 60 µg cm^−2^).

The graph demonstrates that the addition of NiO_x_ to the Fe_2_O_3_ photoanode enhances the photocurrent density across the potential range tested. However, the relationship deviates from linearity. While increasing the NiO_x_ loading from 2 µg cm^−2^ to 30 µg cm^−2^ leads to a notable increase in photocurrent density, a further increase to 60 µg cm^−2^ results in a slight decrease. This suggests an optimal loading range for NiO_x_ exists to maximize the photoelectrochemical performance. Exceeding this optimum loading may lead to detrimental effects such as blocking active sites or hindering charge transfer processes. The data suggests that an intermediate loading of NiO_x_ provides the best enhancement of photocurrent density.

Figure 11b shows the relationship between NiO_x_ loading percentage and photocurrent density on a Fe_2_O_3_ + NiO_x_ photoelectrode at a fixed potential of 0.202 V vs. Ag/AgCl. The graph indicates an optimal NiO_x_ loading exists, resulting in peak photocurrent density. At low NiO_x_ loadings, the photocurrent density increases as more catalytic sites become available. However, beyond a certain point (approximately 20–30%), the photocurrent density begins to decrease. This suggests that excessive NiO_x_ loading may hinder charge transfer or create detrimental effects, such as increased electron-hole recombination or light scattering, thereby reducing the overall efficiency of the photoelectrochemical process. The data suggests there’s a volcano-shaped relationship between NiO_x_ loading and performance, indicating an optimum loading range for maximized photocurrent generation (16 µg cm^−2^ of NiO_x_).

**Figure 12:**
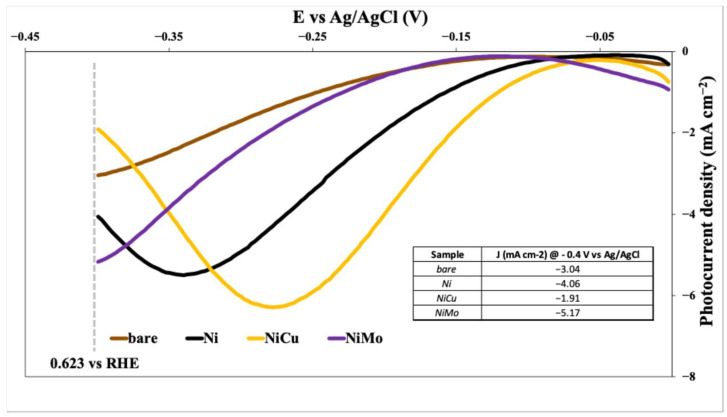
Photocurrent density vs. applied potential (vs. Ag/AgCl) for CuO photocathodes modified with Ni, NiCu, and NiMo co-catalysts. All data collected under 1 SUN AM 1.5 illumination in 1 M KOH with a catalyst loading of 16 µg cm^−2^.

An electrochemical comparison, in half-cell configuration, was made among different Ni-based co-catalysts deposited on the cupric oxide (CuO). For the half-cell electrochemical characterization, by scanning the potential from −0.4 V to 0 V vs. Ag/AgCl, the following results were obtained (Figure 12), reported as the difference between the current density measured in the light and the current density measured in the dark.

Figure 12 presents a comparative analysis of the photoelectrochemical performance of a CuO photocathode modified with different nickel-based co-catalysts: Ni, NiCu, and NiMo (all with a catalyst loading of 16 µg cm^−2^). The data, obtained from half-cell electrochemical measurements, plots photocurrent density (mA/cm^2^) against applied potential (V vs. Ag/AgCl).

Several key observations can be made:

- Significant Enhancement: All three co-catalysts (Ni, NiCu, and NiMo) demonstrably improve the photocurrent density of the CuO photocathode compared to the bare CuO electrode. This indicates that these materials effectively enhance the hydrogen evolution reaction (HER) kinetics.
- NiMo Superiority: Among the tested materials, NiMo provided the highest enhancement in photocurrent density over the entire potential range, confirming the strong synergistic interaction between Ni and Mo in promoting HER activity. This superior performance likely stems from optimized electronic properties and/or enhanced catalytic activity at the co-catalyst-electrolyte interface.
- NiCu Performance: The NiCu co-catalyst also demonstrates a noticeable improvement over the bare CuO and the Ni-only co-catalyst, although it falls short of NiMo’s performance. This indicates that the copper addition positively influences HER activity, but possibly less than the combined effects of nickel and molybdenum.
- Potential-Dependent Behavior: The photocurrent density is strongly potential-dependent for all samples. At more negative potentials, the HER is favored, and thus, photocurrent density increases. The specific shapes of the curves may reveal information about the charge transfer mechanisms and reaction kinetics for each co-catalyst.

The table embedded in the graph quantifies these observations by listing the photocurrent density values at −0.4 V vs. Ag/AgCl for each sample.

**Figure 13:**
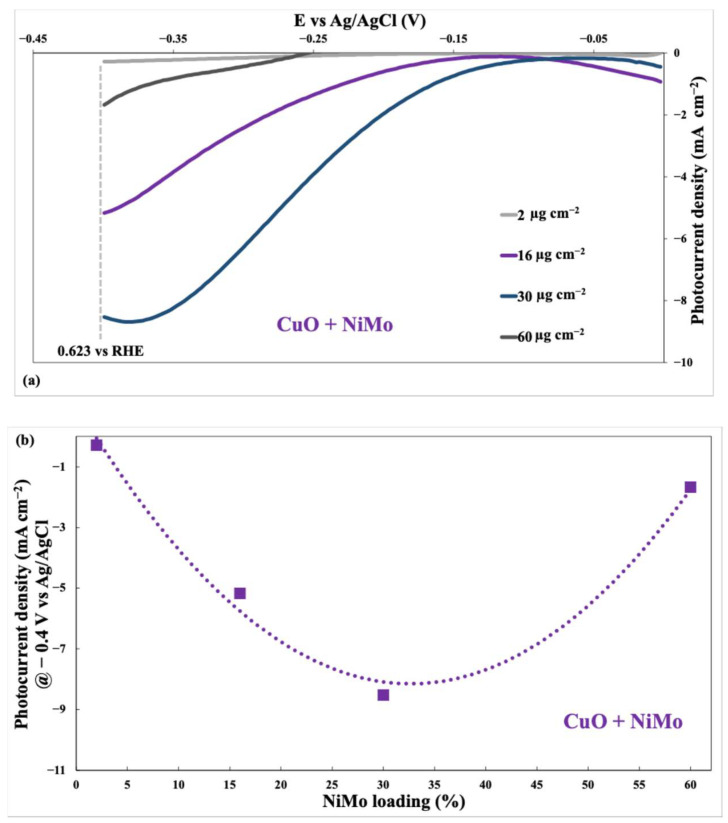
(**a**) Polarization curves of CuO photocathodes modified with different NiMo loadings (2–60 µg cm^−2^); (**b**) Volcano-type relationship of photocurrent density vs. NiMo loading at −0.4 V vs. Ag/AgCl in 1 M KOH under 1 SUN illumination.

Figure 13a displays the photocurrent density versus applied potential (vs. Ag/AgCl) for CuO photocathodes modified with various loadings of NiMo co-catalyst (2 µg cm^−2^, 16 µg cm^−2^, 30 µg cm^−2^, and 60 µg cm^−2^).

The figure shows that increasing the NiMo loading generally leads to a higher photocurrent density, particularly in the potential range around −0.3 V vs. Ag/AgCl. This indicates that the NiMo co-catalyst enhances the hydrogen evolution reaction (HER) on the CuO surface. However, the relationship between NiMo loading and photocurrent density is not strictly linear; the increase is more pronounced at lower loadings. The highest photocurrent density does not occur at the highest NiMo loading, suggesting that an optimum loading exists. Loading beyond this optimum likely leads to negative effects, such as blocking of active sites or hindering charge transfer. In summary, Figure 13a qualitatively demonstrates that NiMo acts as an effective co-catalyst for HER on CuO, with an optimal loading range.

Figure 13b illustrates the relationship between NiMo loading (in µg cm^−2^) on a CuO photocathode and the resulting photocurrent density at a specific potential (−0.4 V vs. Ag/AgCl) in a 1 M KOH electrolyte under illumination.

The graph exhibits a pronounced volcano-type trend, highlighting the existence of an optimal NiMo loading that maximizes photocurrent density. At low NiMo loadings, the photocurrent density increases as more NiMo is added. This is expected because the NiMo co-catalyst enhances the hydrogen evolution reaction (HER) at the CuO surface. However, beyond a certain loading, further addition of NiMo leads to a decrease in photocurrent density. This decrease at higher loadings suggests a trade-off between the beneficial catalytic effect of NiMo and potentially detrimental factors such as:

- Blocking of active sites. Excessive NiMo could cover active sites on the CuO surface, reducing the number of sites available for HER.
- Increased charge transfer resistance. A thick layer of NiMo might hinder efficient charge transfer between the CuO and the electrolyte.
- Light scattering/absorption. A high NiMo loading could scatter or absorb light, reducing the amount of light reaching the CuO and decreasing the photocurrent generation.

The optimal loading appears to be around 30 µg cm^−2^ as this corresponds to the peak photocurrent density.

### 3.6. ECSA Results

Table 3 and Table 4 summarize C_dl_ and ECSA values for photoanodes and photocathodes.

## 4. Discussion

The following sections critically analyze the effects of co-catalyst composition and loading on the PEC performance of α-Fe_2_O_3_ photoanodes and CuO photocathodes. Comparisons with literature are included to contextualize the experimental findings, and to highlight the relevance of catalyst engineering strategies for tandem PEC systems based on earth-abundant materials.

### 4.1. Anodic Co-Catalysts: Hematite Photoanode

The application of Ni-based co-catalysts to hematite photoanodes has been widely studied for improving charge-transfer kinetics and surface OER activity. In our study, the NiO_x_-modified hematite exhibited the highest photocurrent density, surpassing 0.8 mA/cm^2^ at 0.2 V vs. Ag/AgCl, in line with literature reporting the beneficial role of Ni-based surface layers for OER on iron oxides [24,25]. By contrast, at the same potential NiFeO_x_ and NiWO_4_ yield photocurrents lower than the bare electrode, i.e., they do not provide an improvement under our operating bias.

XPS survey spectra (Figure S15) confirm the expected surface elements for the three oxides (Ni and O for NiO_x_; Ni, Fe and O for NiFeO_x_; Ni, W and O for NiWO_4_), without extraneous signals; while the survey does not allow a full oxidation-state deconvolution, the presence of oxidic environments on NiO_x_ is consistent with the formation, under anodic polarization, of Ni(OH)_2_/NiOOH-like redox centers known to facilitate hole transfer to the electrolyte. This interpretation is supported by EIS measurements at 0.20 V vs. Ag/AgCl under 1 SUN illumination, where NiO_x_ displays the smallest semicircle diameter among the anodic configurations (lowest R_ct_; Figure S16).

NiFeO_x_, while less effective than NiO_x_ in our experiments, has shown in the literature synergistic effects between Ni and Fe that can enhance conductivity and catalytic efficiency (e.g., Gong et al. [31]); however, the absence of sharp Fe-related peaks in XRD here suggests atomic-scale dispersion, which may limit its available catalytic surface. NiWO_4_, although structurally stable and highly crystalline, showed moderate activity, in agreement with Mancheva et al. [32], where performance was constrained by relatively poor electrical conductivity despite good chemical stability.

Across Fe_2_O_3_ photoanodes, the NiO_x_ co-catalyst delivers the largest ECSA and the highest photocurrent, accompanied by the lowest charge-transfer resistance in EIS. NiFeO_x_ and NiWO_4_ exhibit measurable ECSA but do not outperform the bare electrode at 0.20 V vs. Ag/AgCl. This alignment between ECSA, J–E, and EIS indicates that the density and quality of electrochemically addressable sites is a primary driver of interfacial OER kinetics on hematite. Mechanistically, the formation of Ni(OH)_2_/NiOOH-like surface phases under anodic bias supplies redox-active centers that facilitate hole transfer to the electrolyte; Fe incorporation (NiFeO_x_) may further tune the electronic structure and proton-coupled electron-transfer steps, but in our conditions it does not translate into a current gain. NiWO_4_ may improve wettability and interfacial contact relative to the bare substrate, yet its lower electroactive area and less favorable charge-transfer characteristics explain the more modest performance. Overall, at 0.20 V vs. Ag/AgCl the ranking is NiO_x_ > bare > (NiFeO_x_ ≈ NiWO_4_).

Consistently with the electrochemical trends, BET/PSD analysis (Section 3.3, Table 1) shows that the oxide co-catalysts are mesoporous (type-IV, H3/H4 hysteresis), with NiOₓ exhibiting the largest specific surface area and pore volume (S_BET ≈ 56 m^2^ g^−1^; V_p ≈ 0.12 cm^3^ g^−1^; D_p ≈ 7.8 nm), followed by NiFeO_x_ (38; 0.09; 8.5 nm) and NiWO_4_ (22; 0.06; 10.2 nm). These textural descriptors align with the ECSA ranking (Table 3) and help rationalize the superior photocurrent of NiO_x_-modified hematite. At the same time, the fact that NiFeO_x_ and NiWO_4_ do not outperform the bare electrode at 0.20 V vs. Ag/AgCl indicates that geometric surface area alone is not sufficient: surface chemistry and interfacial kinetics remain decisive. In particular, the Ni(OH)_2_/NiOOH-like environments inferred by XPS on NiO_x_ and the lower Rct observed by EIS (Figure S16) point to faster hole transfer to the electrolyte, whereas NiWO_4_ is limited by less favorable charge-transfer characteristics, and NiFeO_x_—despite its mesoporosity—does not translate its area into a current gain under our bias. Overall, the BET data reinforce a picture in which mesoporosity facilitates wetting and site accessibility, but the formation of active NiOOH-type surface phases is the key lever for OER on hematite.

### 4.2. Cathodic Co-Catalysts: CuO Photocathode

CuO-based photocathodes benefit significantly from surface modification due to their narrow band gap and strong absorption in the visible range but suffer from charge recombination and poor HER kinetics. Among all cathodic co-catalysts, NiMo achieved the highest photocurrent densities, confirming its excellent catalytic properties for the hydrogen evolution reaction (HER), in agreement with findings by McKone et al. [33], who showed that NiMo alloys exhibit exceptional HER activity in alkaline media, due to the synergistic electronic interactions between nickel and molybdenum. The performance trend NiMo > NiCu > Ni is also consistent with the catalytic hierarchy reported by Angeles-Olvera et al. [23], where bimetallic systems showed enhanced electrocatalytic activity due to optimized adsorption energies of hydrogen intermediates and increased electrochemically active surface area. CuO + NiCu electrodes displayed intermediate performance. This observation reflects similar trends reported by Lo Vecchio et al. [22], in which the incorporation of Cu into Ni enhanced catalytic conductivity while maintaining compatibility with the CuO substrate. This trend is corroborated by EIS measurements performed at −0.4 V vs. Ag/AgCl under 1 SUN illumination. As shown in Figure S17, the NiMo sample exhibits the smallest semicircle diameter, indicating the lowest charge-transfer resistance. Bare CuO and Ni samples show intermediate behavior, while NiCu displays the highest semicircle, confirming the interfacial charge-transfer hierarchy.

For the CuO-based photocathodes, the NiMo sample exhibits survey signals of Ni and Mo, with no detectable O 1 s peak, indicating a predominately metallic surface environment composed of Ni and Mo. The bimetallic Ni–Mo combination enhances HER activity by leveraging Ni’s electrical conductivity and Mo’s ability to adjust hydrogen adsorption energetics, resulting in the superior photocathodic performance observed for NiMo-modified electrodes. In comparison, both metallic Ni and the NiCu alloy also display purely metallic signatures (Ni 2p and Cu 2p respectively), with no significant O signals—these simpler metallic surfaces correlate with their lower catalytic activity for hydrogen evolution relative to NiMo.

On CuO photocathodes, NiMo shows the highest ECSA, the best HER photoresponse, and the smallest EIS semicircle, followed by NiCu, then Ni, with bare CuO performing worst. The close correspondence among ECSA, photocurrent, and charge-transfer resistance indicates that enlarging the electrochemically accessible interface is central to accelerating HER at the CuO/electrolyte boundary. The Ni–Mo synergy is known to stabilize H-adsorption at near-optimal binding strength while maintaining good electronic conductivity, which rationalizes its superior interfacial kinetics. NiCu provides intermediate behavior, consistent with enhanced conductivity and modified hydrogen binding compared to Ni alone. Survey XPS for these metallic co-catalysts is dominated by the Ni and (where applicable) Mo/Cu signals, with only minimal oxygen contributions, in line with their composition. Taken together, the co-catalyst order NiMo > NiCu > Ni > bare is simultaneously captured by ECSA, JV, and EIS, underscoring that HER performance in our CuO system is primarily controlled by the availability of active interfacial sites and their associated charge-transfer pathways.

In contrast to the oxides, BET results for the metallic/alloy powders indicate essentially non-porous textures (Table 2), so the performance order NiMo > NiCu > Ni cannot be attributed to geometric area. Rather, it reflects intrinsic electronic/catalytic effects at the CuO at electrolyte interface: the Ni–Mo synergy optimizes H-adsorbate energetics while maintaining good conductivity, which is consistent with the lowest R_ct_ observed by EIS at −0.4 V vs. Ag/AgCl (Figure S17) and with the highest ECSA within this series (Table 4). Small differences among Kr-BET values (0.5–0.8 m^2^ g^−1^) lie near the resolution limit for low-area metals and should not be over-interpreted. Hence, for CuO photocathodes, textural descriptors carry little predictive power, and interfacial kinetics/electronic structure dominate the HER response, especially at optimal NiMo loadings.

### 4.3. Effect of Co-Catalyst Loading

Both anodic and cathodic systems displayed a volcano-type dependence of photocurrent response on co-catalyst loading. This behavior, common in PEC and electrocatalytic systems, reflects the trade-off between catalytic enhancement and detrimental phenomena such as site blocking, excessive light absorption, and increased charge transfer resistance. Similar behavior has been reported [18], where overloading Ni-based catalysts on photoelectrodes led to a decline in performance due to thick layers acting as recombination centers or light-scattering barriers. Our results confirm that moderate catalyst loadings (16–30 µg cm^−2^) optimize the balance between surface activity and optical/electronic properties, making them ideal for tandem PEC configurations.

Overall, the correlation between co-catalyst composition, loading, and interfacial charge transfer resistance is further supported by EIS analysis (Figures S16 and S17). These Nyquist plots demonstrate that samples with the highest photoelectrochemical performance also display the lowest semicircle diameters, indicating reduced charge-transfer resistance at the electrode–electrolyte interface. This trend confirms that the enhancement in PEC activity is primarily governed by improved interfacial kinetics rather than solely by increased surface coverage or light absorption. In particular, the beneficial effects of NiO_x_ and NiMo are attributed to their favorable electronic structures, which lower the overpotential for HER and OER, respectively, and accelerate interfacial charge separation.

In addition, the volcano-type trends observed in Figure 11b and Figure 13b emphasize the existence of an optimal co-catalyst loading range, typically between 16 and 30 µg cm^−2^. Below this threshold, the number of available active sites may be insufficient to significantly promote charge transfer, while excessive loading can introduce light-blocking effects, increased recombination centers, or hinder charge transport within the catalyst layer. These findings align with literature reports on non-linear activity–loading relationships in nickel-based catalysts and reinforce the importance of rational co-catalyst engineering for high-efficiency PEC devices.

## 5. Conclusions

Nickel-based co-catalysts were effectively integrated onto α-Fe_2_O_3_ photoanodes and CuO photocathodes, resulting in enhanced photoelectrochemical (PEC) performance. NiO_x_ led to the highest photocurrent improvement on hematite, while NiMo was the most effective on CuO, confirming the critical role of composition in promoting OER and HER kinetics. In both cases, an optimal loading was identified, beyond which performance declined due to charge transfer limitations and light attenuation. These results confirm the relevance of co-catalyst engineering in improving charge separation and surface kinetics, offering a cost-effective approach for scalable, earth-abundant PEC hydrogen production.

Finally, to validate practical viability, durability will be evaluated in future work using full Fe_2_O-CuO tandem cells under medium/long-duration operation (galvanostatic/potentiostatic) with light/dark cycling and accelerated stress, followed by post-mortem structural and surface analyses. These tests will quantify stability under device-relevant conditions beyond the short half-cell polariations adopted here.

## Figures and Tables

**Table 1:** Principal gas-adsorption measurement descriptors for photoanodic co-catalysts.

Photoanode	Specific Surface Area (m^2^ g^−1^)	Pore Volume (cm^3^ g^−1^)	Modal Pore Diamater (nm)
NiO_x_	56	0.12	7.8
NiFeO_x_	38	0.09	8.5
NiWO_4_	22	0.06	10.2

**Table 2:** Principal gas-adsorption measurement descriptors for photocathodic co-catalysts.

Photocathode	Specific Surface Area (m^2^ g^−1^)	Pore Volume (cm^3^ g^−1^)
Ni	0.6	<0.002
NiCu	0.8	<0.003
NiMo	0.5	<0.002

**Table 3:** C_dl_ and ECSA calculated values for photoanodes.

Photoanode	C_dl_ (mF cm^−2^)	ECSA (cm^2^)
bare	0.6	15
NiO_x_	2.4	60
NiFeO_x_	1.6	40
NiWO_4_	1	25

**Table 4:** C_dl_ and ECSA calculated values for photocathodes.

Photocathode	C_dl_ (mF cm^−2^)	ECSA (cm^2^)
bare	0.8	20
Ni	1.2	30
NiCu	1.8	45
NiMo	3	75

## Data Availability

The datasets obtained and analyzed during the current study are available from the corresponding author upon reasonable request.

## Supplementary Materials

The following supporting information can be downloaded at: https://www.mdpi.com/article/10.3390/nano15201551/s1. Figures S1–S5: Synthesis schemes of Ni, NiOx, NiFeOx, NiWO_4_, NiCu, and NiMo co-catalysts. Figure S6: Reactor scheme used for half-cell electrochemical characterization: (a) photoanode setup and (b) photocathode setup. Figures S7–S12: XRF spectra of all co-catalysts (NiOx, NiFeOx, NiWO_4_, Ni, NiCu, NiMo), showing Kα and Kβ emission lines. Figure S13: TEM-EDX analysis of (a) NiFeOx, (b) NiWO_4_, (c) Ni, (d) NiCu, and (e) NiMo. Figure S14: SEM-EDX analysis of (a) NiOx, (b) NiFeOx, (c) NiWO_4_, (d) Ni, (e) NiCu, and (f) NiMo. Figure S15: XPS survey spectrum of the co-catalysts. Figure S16: EIS spectra of Fe_2_O_3_ photoanodes with NiOx, NiFeOx, and NiWO_4_ at 0.2 V vs. Ag/AgCl under 1 SUN. Figure S17: EIS spectra of CuO photocathodes with Ni, NiCu, and NiMo at −0.4 V vs. Ag/AgCl under 1 SUN.
