# Supplementary material for: Experimental Investigation of Nickel-Based Co-Catalysts for Photoelectrochemical Water Splitting Using Hematite and Cupric Oxide Nanostructured Electrodes
Source: Nanomaterials (Basel). 2025 Oct 11;15(20):1551. doi: 10.3390/nano15201551 (PMC12566993; doi:10.3390/nano15201551)
Supplement: Supplementary file 1 [file nanomaterials-15-01551-s001.zip › nanomaterials-3888739-supplementary.pdf]

nanomaterials

MDPI

SUPPLEMENTARY INFORMATION

Experimental Investigation of Nickel-Based Co-Catalysts for Photoelectrochemical Water Splitting Using Hematite and Cupric Oxide Nanostructured Electrodes

Maria Aurora Mancuso [1,†], Rossana Giaquinta [1,†], Carmine Arnese [1], Patrizia Frontera [2], Anastasia Macario [3], Angela Malara [2] and Stefano Trocino [1,*]

[1] Institute for Advanced Energy Technologies "Nicola Giordano" CNR-ITAE, Via Salita S. Lucia Sopra Contesse, 5, 98126 Messina, Italy
[2] Department of Civil, Energy, Environmental and Material Engineering, Mediterranea University of Reggio Calabria, 89124 Reggio Calabria, Italy[3] Dipartimento di Ingegneria per l'Ambiente, Università della Calabria, I-87036 Arcavacata di Rende, CS, Italy
* Correspondence: stefano.trocino@cnr.it; Tel.: +39-090-624-270
† These authors contributed equally to this work.

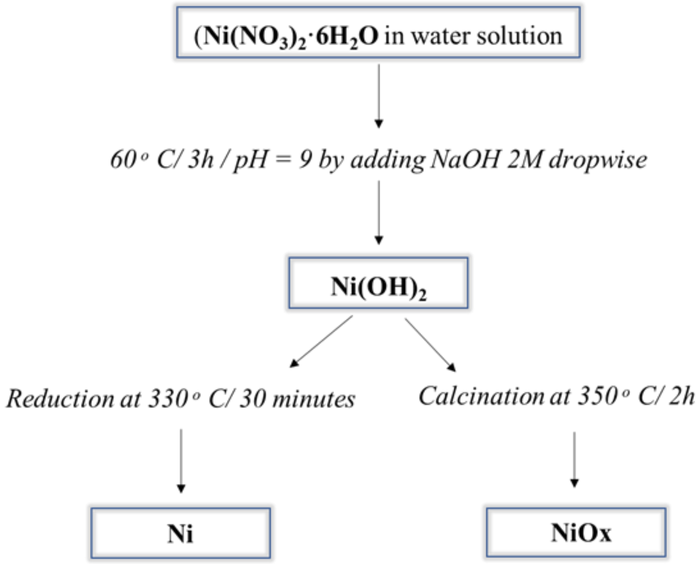

Figure S1. Synthesis scheme for Ni and NiOx, used respectively as cathodic and anodic co-catalysts.

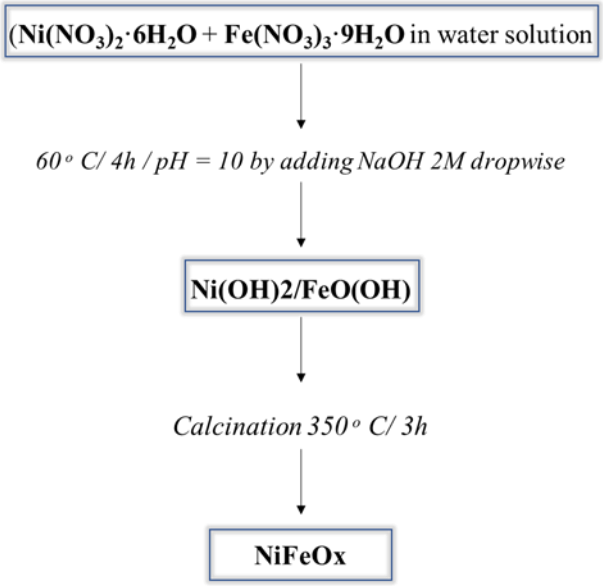

Figure S2. Synthesis scheme for NiFeOx anodic co-catalyst.

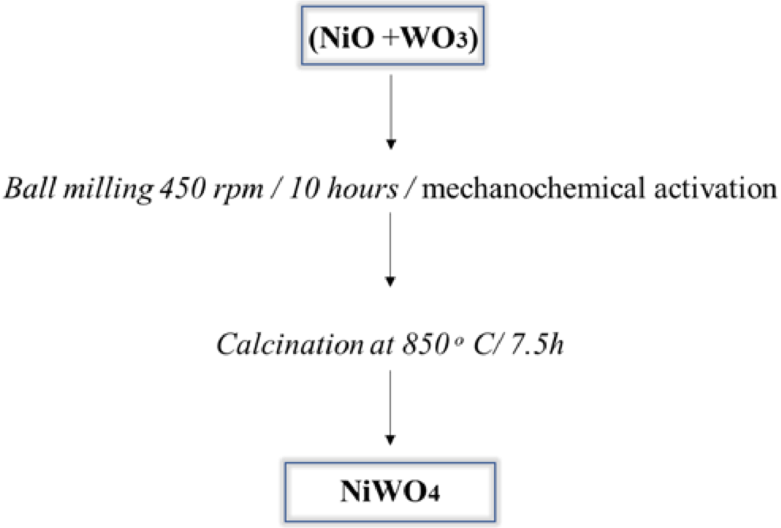

Figure S3. Synthesis scheme for $NiWO_4$ anodic co-catalyst.

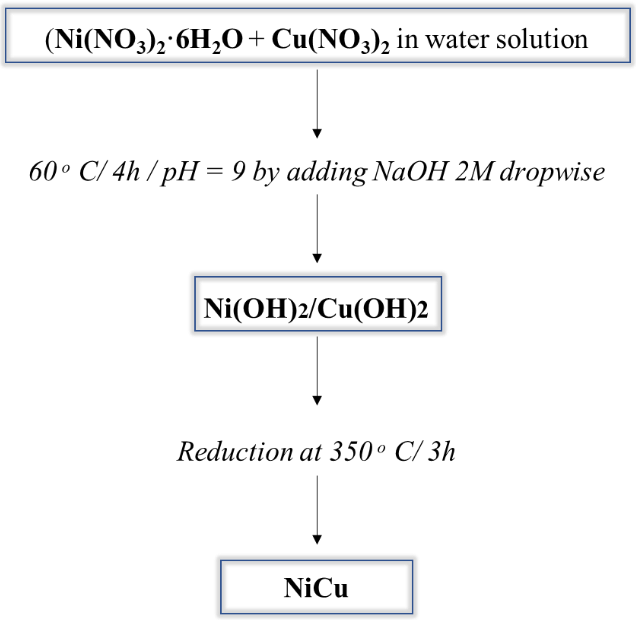

Figure S4. Synthesis scheme for NiCu cathodic co-catalyst.

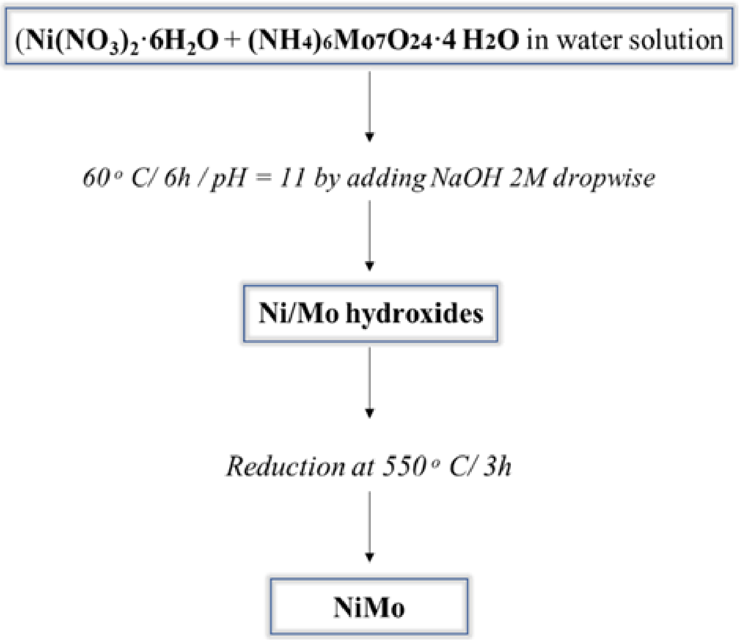

Figure S5. Synthesis scheme for NiMo cathodic co-catalyst.

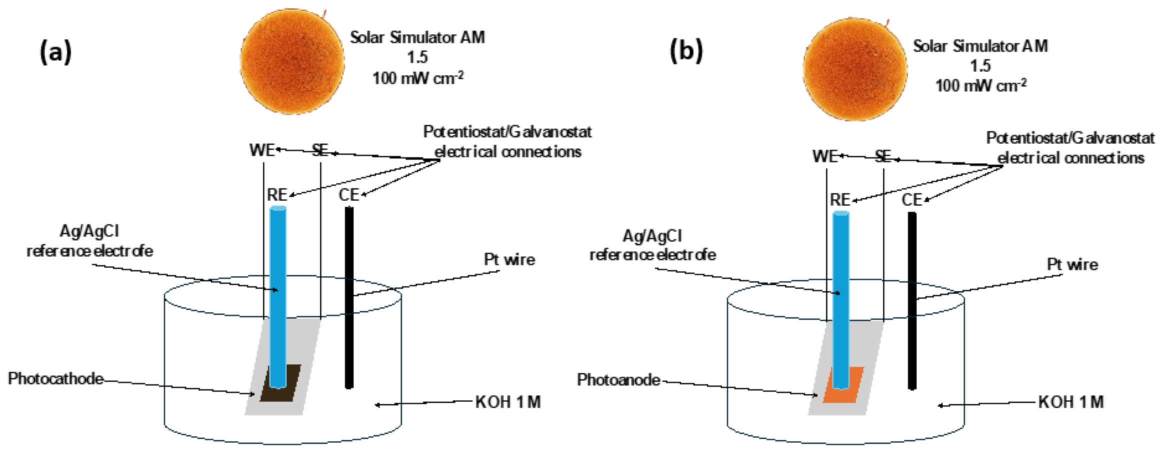

Figure S6. Reactor scheme used for half-cell electrochemical characterization: (a) photoanode setup, (b) photocathode setup.

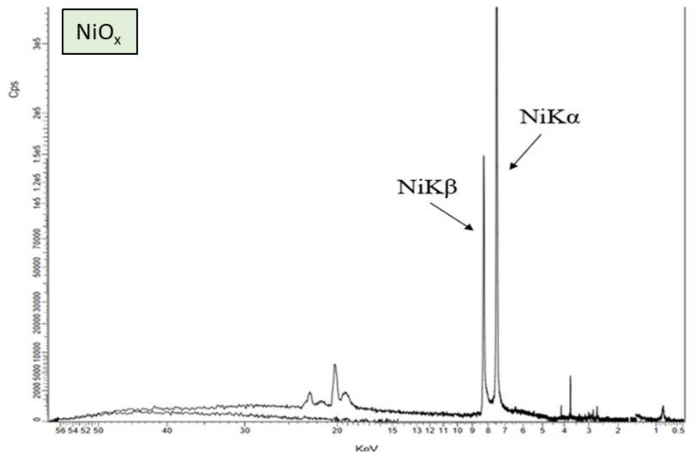

Figure S7. XRF spectrum showing Kα and Kβ emission lines of Ni for the NiOx sample.

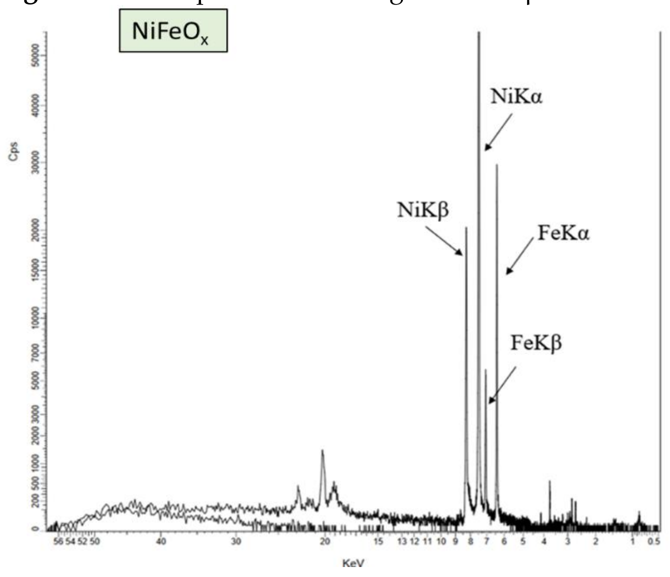

Figure S8. XRF spectrum with Kα and Kβ emission lines of Ni and Fe for the NiFeOx sample.

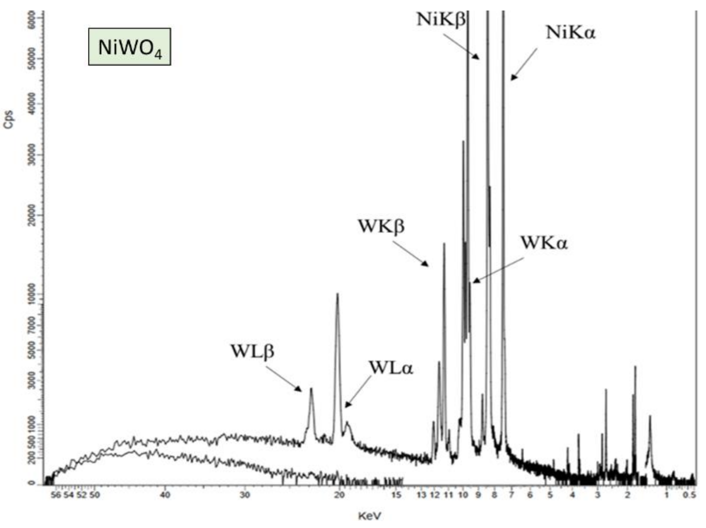

Figure S9. XRF spectrum with Kα and Kβ emission lines of Ni, and Kα, Kβ, Lα, and Lβ lines of W for the $NiWO_4$ sample.

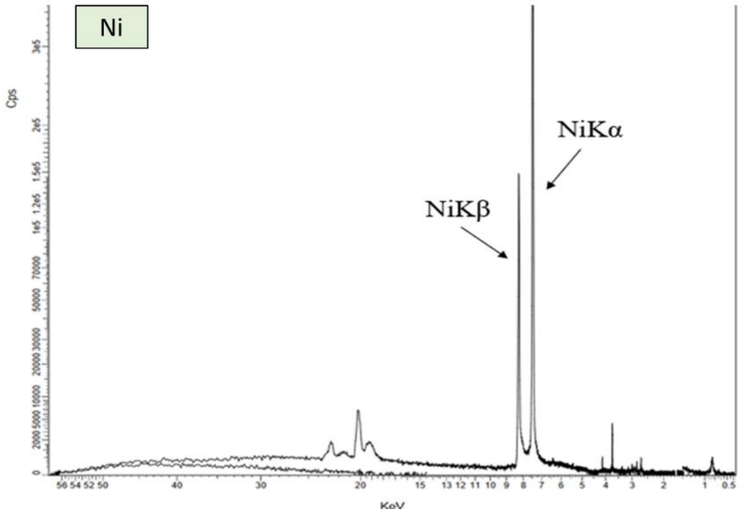

Figure S10. XRF spectrum of Ni cathodic co-catalyst showing Kα and Kβ emission lines of Ni.

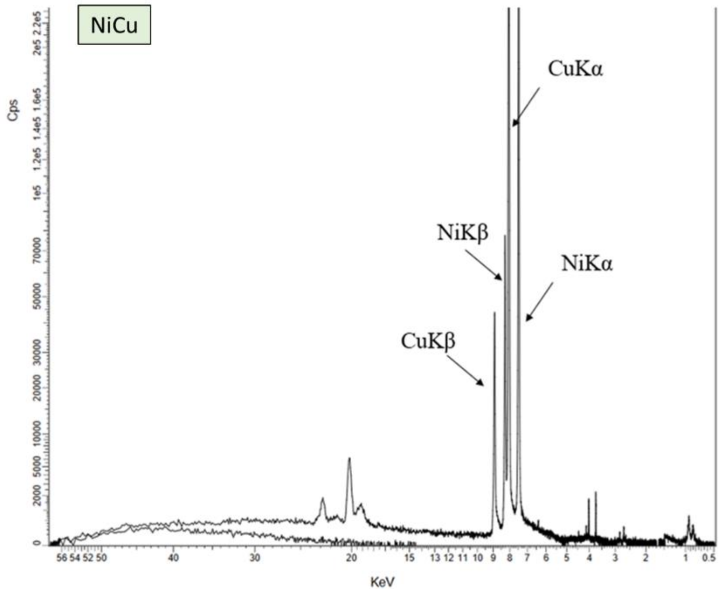

Figure S11. XRF spectrum of NiCu alloy showing Kα and Kβ emission lines of both Ni and Cu.

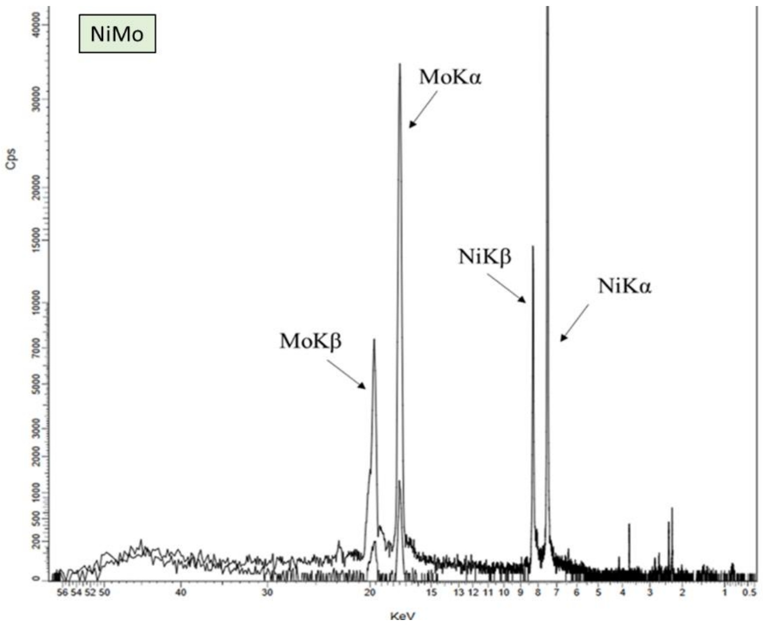

Figure S12. XRF spectrum of NiMo co-catalyst showing Kα and Kβ emission lines of Ni and Mo.

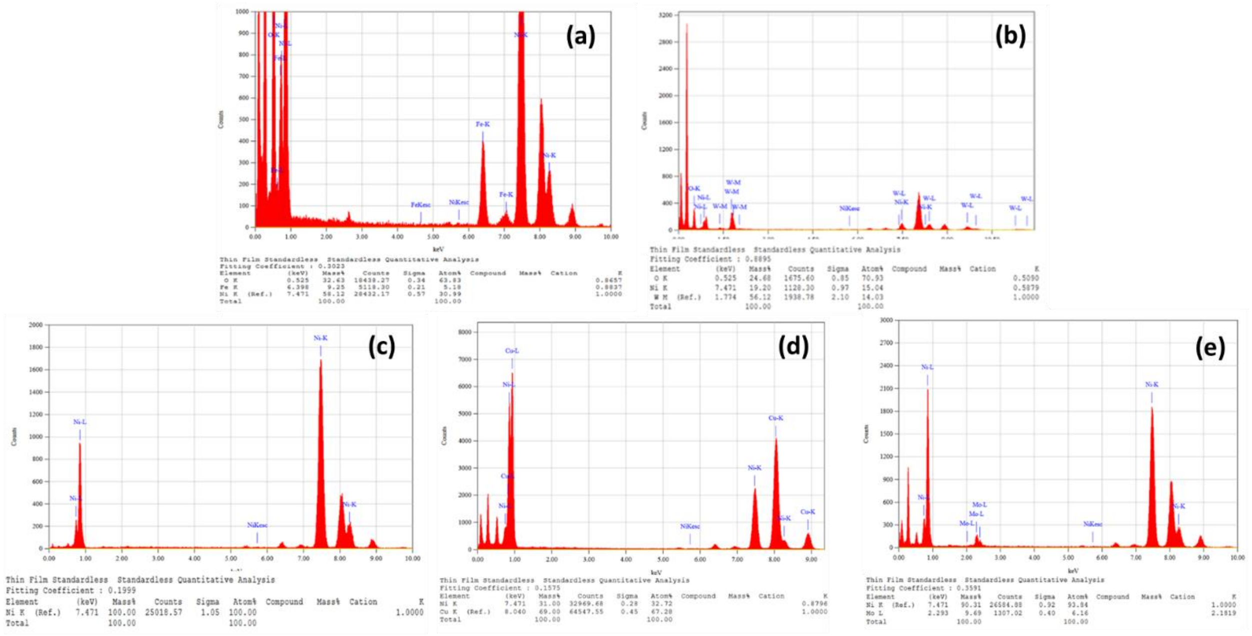

Figure S13. TEM-EDX analysis of: (a) NiFeOx, (b) $NiWO_4$ as anodic co-catalysts; (c) Ni, (d) NiCu, (e) NiMo as cathodic co-catalysts.

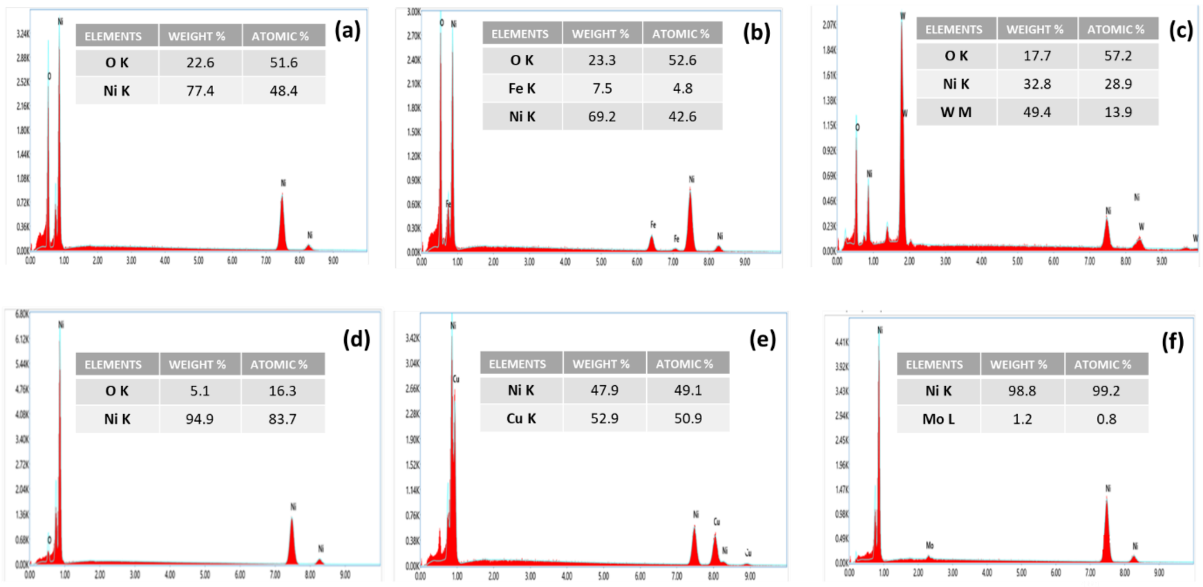

Figure S14. SEM-EDX analysis of: (a) NiOx, (b) NiFeOx, (c) $NiWO_4$ as anodic co-catalysts; (d) Ni, (e) NiCu, (f) NiMo as cathodic co-catalysts.

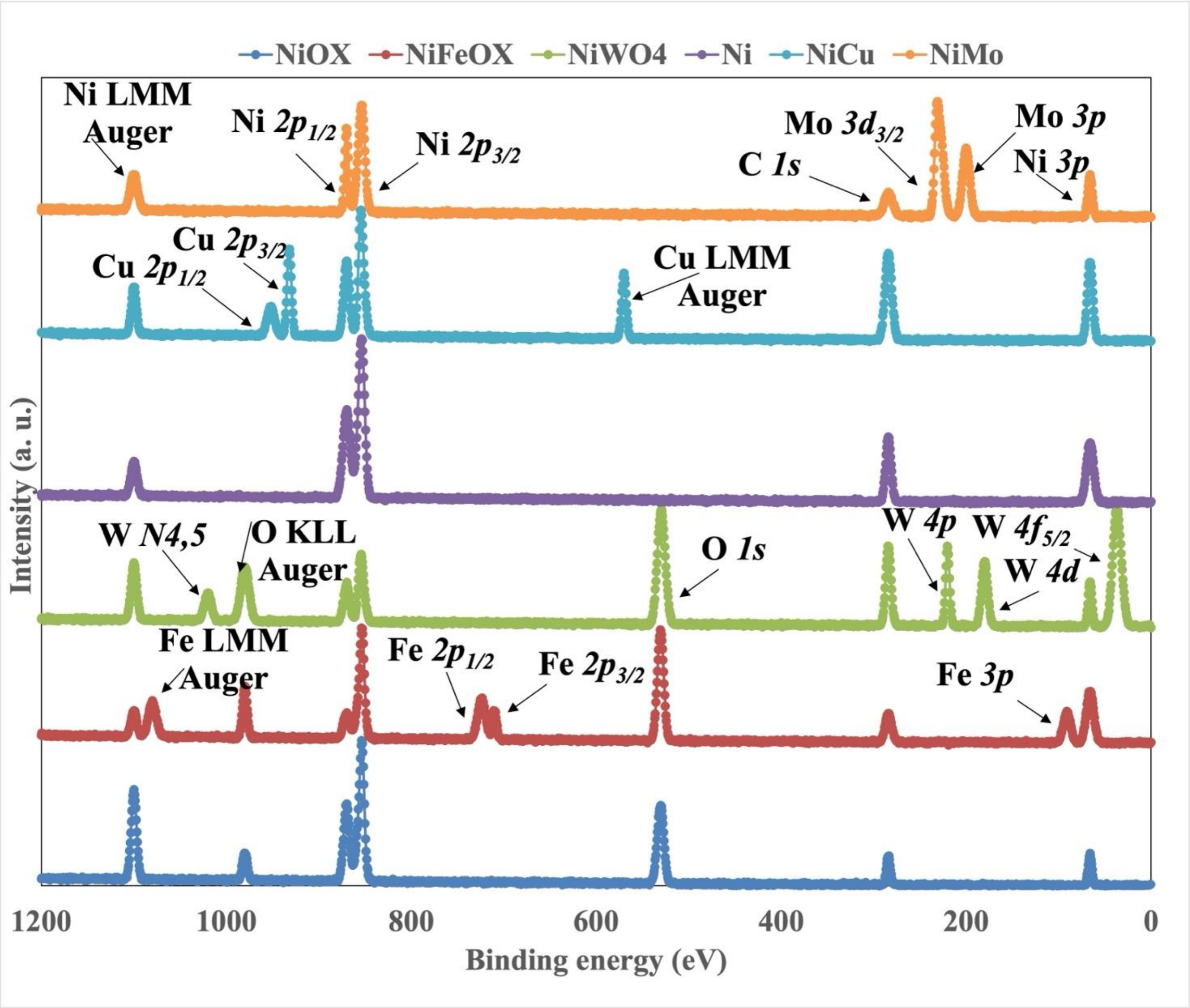

Figure S15. X-ray Photoelectron Spectroscopy (XPS) survey spectra of the investigated co-catalysts: (a) NiO_x, (b) $NiFeO_x$, (c) $NiWO_4$, (d) Ni, (e) NiCu, and (f) NiMo. Peaks corresponding to the expected

elements are identified for each sample, including Ni, O, and C (adventitious carbon) in all spectra, together with Fe ($NiFeO_x$), W ($NiWO_4$), Cu (NiCu), and Mo (NiMo). These data confirm the surface composition of the materials and the absence of extraneous elements, providing qualitative evidence of the targeted surface chemistries that support the proposed catalytic roles of the different co-catalysts.

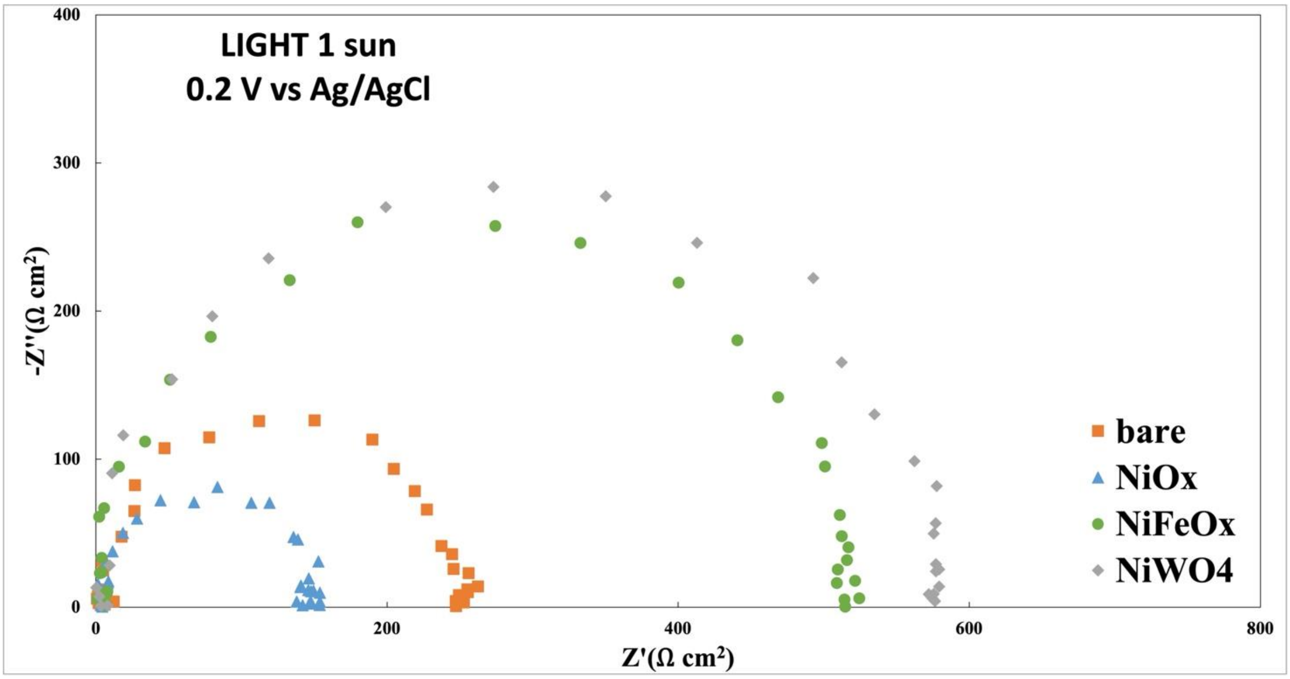

Figure S16. EIS spectra of Fe_2O_3 photoanodes measured at 0.2 V vs Ag/AgCl in 1 M KOH under 1 SUN illumination. The data compare the bare Fe_2O_3 electrode with samples modified by NiO_x, $NiFeO_x$, and $NiWO_4$ co-catalysts. A clear reduction in the semicircle diameter is observed for the NiO_x-modified sample, indicating significantly lower charge-transfer resistance. $NiFeO_x$ and $NiWO_4$ electrodes display larger diameters, consistent with slower interfacial kinetics and reduced PEC performance.

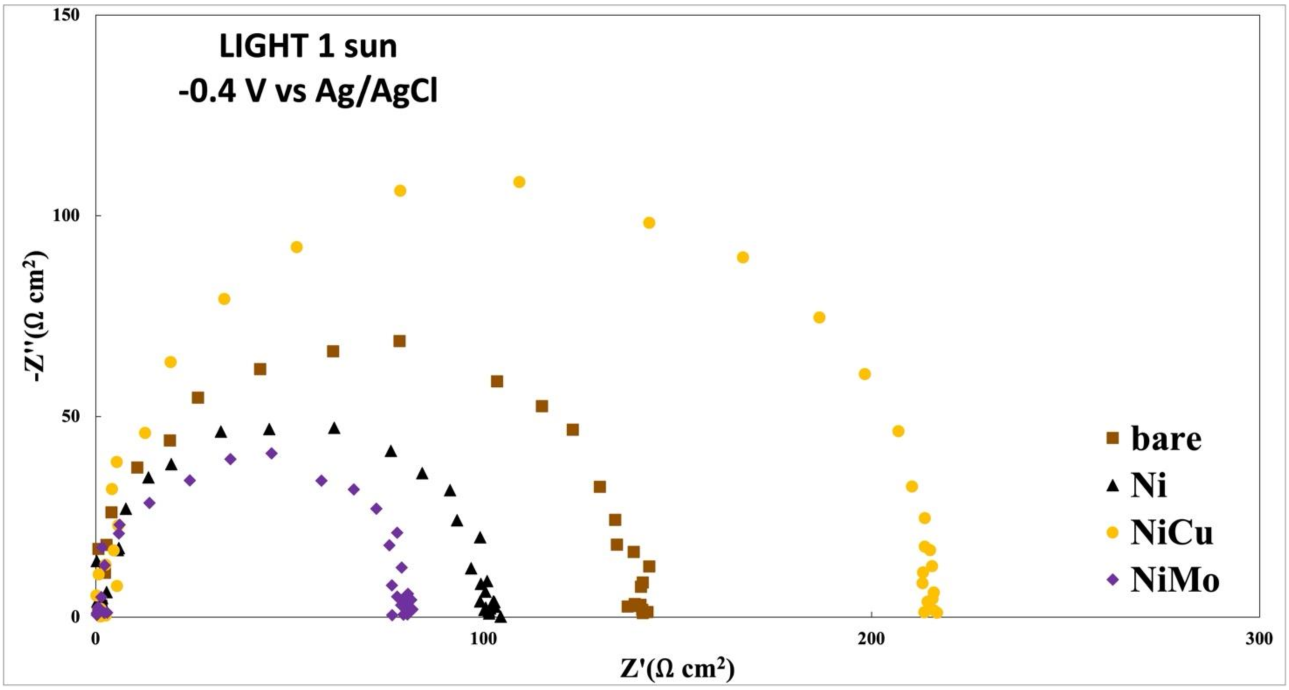

Figure S17. EIS spectra of CuO photocathodes measured at –0.4 V vs Ag/AgCl in 1 M KOH under 1 SUN illumination. The data compare the bare CuO electrode with samples modified by Ni, NiCu, and NiMo co-catalysts. A clear reduction in the semicircle diameter is observed for the NiMo-modified sample, indicating significantly lower charge-transfer resistance. Ni and NiCu electrodes display larger diameters, consistent with slower interfacial kinetics and reduced PEC performance.
